# Depletion of *Plasmodium berghei* Plasmoredoxin Reveals a Non-Essential Role for Life Cycle Progression of the Malaria Parasite

**DOI:** 10.1371/journal.pone.0002474

**Published:** 2008-06-25

**Authors:** Kathrin Buchholz, Stefan Rahlfs, R. Heiner Schirmer, Katja Becker, Kai Matuschewski

**Affiliations:** 1 Interdisciplinary Research Centre, Justus-Liebig University, Giessen, Germany; 2 Biochemistry Centre, Ruprecht-Karls University, Heidelberg, Germany; 3 Department of Parasitology, School of Medicine, Heidelberg University, Heidelberg, Germany; Federal University of São Paulo, Brazil

## Abstract

Proliferation of the pathogenic *Plasmodium* asexual blood stages in host erythrocytes requires an exquisite capacity to protect the malaria parasite against oxidative stress. This function is achieved by a complex antioxidant defence system composed of redox-active proteins and low MW antioxidants. Here, we disrupted the *P. berghei* plasmoredoxin gene that encodes a parasite-specific 22 kDa member of the thioredoxin superfamily. The successful generation of plasmoredoxin knockout mutants in the rodent model malaria parasite and phenotypic analysis during life cycle progression revealed a non-vital role *in vivo*. Our findings suggest that plasmoredoxin fulfils a specialized and dispensable role for *Plasmodium* and highlights the need for target validation to inform drug development strategies.

## Introduction

*Plasmodium*, the etiologic agent of malaria, is a unicellular facultative intracellular parasite of the phylum *Apicomplexa*. A hallmark of the malaria parasite is its remarkable capacity to specifically invade and replicate inside red blood cells. This intraerythrocytic proliferation phase ultimately leads to the disease known as malaria. Due to the high metabolic rates of the rapidly growing and multiplying parasite, large quantities of toxic redox-active by-products are generated. Additional reactive oxygen and nitrogen species are generated by immune effector cells of the host in response to parasite infection and during hemoglobin degradation in the food vacuole of the parasite. Therefore, inside erythrocytes, the ability of *Plasmodium* to defend itself against oxidative damage is of vital importance for parasite survival 1, 2, and it appears to be highly effective in this respect [3]. *Plasmodium* employs multiple biochemical pathways that mediate antioxidant defense and redox-regulation and play a central role in pathogenesis [4]–[9].

In most eukaryotic organisms, redox-active enzymes, such as catalase, superoxide dismutase, and peroxidases as well as an enzymatic cascade that generates reduced electron donors, i.e. glutathione (GSH) and thioredoxin (Trx), sustain the cellular redox homeostasis [10]. This redox network is split into two major arms, the GSH and the Trx system, that serve complementary functions in antioxidant defense and DNA synthesis. Interestingly, the malarial parasite *Plasmodium* lacks two central antioxidant enzymes: (i) catalase that typically detoxifies hydrogen peroxide and (ii) a classical glutathione peroxidase, a selenoenyzme that reduces lipid hydroperoxides to their alcohols [9]. This apparent deficiency further underscores the central importance of the thioredoxin system in the parasite. In good agreement, Trx reductase, which transfers electrons from NADPH to Trx, appears to perform vital functions for asexual development of the malaria parasite *in vitro* and is considered an attractive target for antimalarial drug development [11].

Intriguingly, malaria parasites possess-in addition to the classical thioredoxins-a *Plasmodium*-specific member of the thioredoxin superfamily termed plasmoredoxin (Plrx) (*P. falciparum* GenBank AAF87222) [12]. Plrx is a 22 kDa dithiol protein with the unique active site sequence WCKYC. Plrx is not reduced by thioredoxin reductase but can react with glutaredoxin and glutathione. Although a non-enzymatic reaction between reduced Trx and glutathione disulfide (GSSG) has been described in insects, which lack glutathione reductase [13], the physiologic electron donor for *P. falciparum* plasmoredoxin still remains to be identified. As described for certain thioredoxins and glutaredoxins, Plrx has been shown to serve as electron donor for ribonucleotide reductase. Furthermore, the protein is capable of reducing disulfide bonds in general and in particular *P. falciparum* thioredoxin peroxidase 1, the major cytosolic peroxiredoxin of the parasite [12], [14].

In this study, we addressed the cellular role of *Plrx* in the rodent malaria model parasite *Plasmodium berghei*. Targeted gene disruption permits drug target validation or elucidation of the *in vivo* role of the gene product. The corresponding predicted experimental outcomes are refractoriness to gene targeting, which would correlate with a vital role in asexual blood stage development, or a detectable phenotype during life cycle progression, respectively. Because of the potential to design tailor-made inhibitors of the *Plasmodium* redox network as innovative antimalarial drugs [4], [5], target validation of individual redox-active enzymes paves the way for future drug discovery approaches. Our findings that loss of *Plrx* function does not affect parasite development under normal growth conditions exemplifies the important role of reverse genetics in guiding drug development against malaria.

## Results

### Generation of *Plrx(-)* parasites

**Figure 1:**
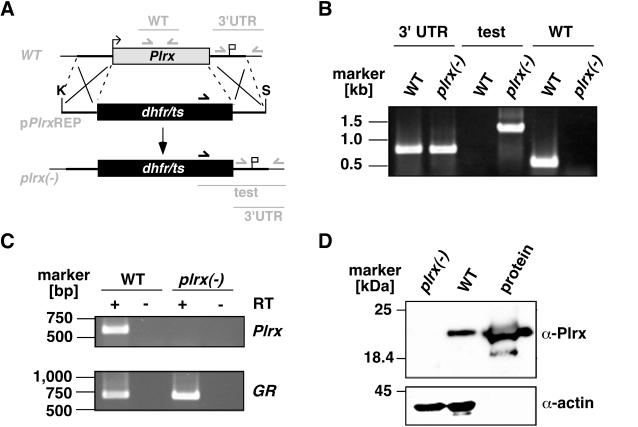
Targeted deletion of the *P. berghei* plasmoredoxin gene. (A) Replacement strategy for targeted gene disruption of *PbPlrx*. The wild-type *Plrx* locus (*WT*) is targeted with a *Kpn*I (K)/ *Sac*II (S)-linearized replacement plasmid (p*Plrx*Rep) containing the 5′and 3′UTR of *PbPlrx* and the positive selection marker *TgDHFR-TS*. After double cross over homologous recombination, the *Plrx* open reading frame is substituted by the selection marker, resulting in the mutant *Plrx(-)* allele. Replacement- and WT-specific test primer combinations and expected fragments are shown as lines. (B) Replacement-specific PCR analysis. Confirmation of the predicted gene targeting is done by primer combinations that only amplify a signal in the recombinant locus (test). The absence of a WT-specific signal in the clonal *Plrx(-)* population confirms the purity of the mutant parasite line. (C) Depletion of *Plrx* transcripts in *Plrx(-)* parasites. cDNA from WT and *Plrx(-)* blood stages was used as template for *Plrx*-specific PCR reactions (upper panel). Amplification of glutathione reductase (*GR*) transcripts was used as a positive control (lower panel). (D) Western blot analysis of WT and *Plrx(-)* blood stages. Extracts from WT or *Plrx(-)* (16 µg total protein each) were separated on a 15% SDS gel and probed with the polyclonal anti-*Plrx* serum (upper panel) or a polyclonal anti-actin serum (lower panel). As a positive control 160 ng recombinantly expressed *P. berghei* plasmoredoxin (protein) was added.

To test whether plasmoredoxin is important for asexual replication of *P. berghei*, we first targeted the *PbPlrx* gene with an integration vector that disrupts the gene locus via a single cross over event. After a single transfection we successfully integrated the disruption plasmid (data not shown). To confirm our unexpected finding that *PbPlrx* can be disrupted we constructed a replacement vector containing the *PbPlrx* 5′ and 3′ UTRs that flank the positive selectable marker, which confers resistance to the antifolate pyrimethamine (Fig. 1A). Upon a double cross over event this vector is predicted to disrupt the entire *PbPlrx* open reading frame (ORF). After continuous selection with oral pyrimethamine a resistant population was obtained and genotyped (data not shown). This parental parasite population contained the correct integration mixed with WT parasites and was used for cloning of four independent parasite *Plrx-*deficient lines, named *Plrx(-)Rep*. Replacement-specific PCR analysis verified the correct replacement event after homologous recombination (Fig. 1B). To confirm the absence of *Plrx* transcripts in *Plrx(-)* parasites, RT-PCR and subsequent cDNA synthesis was performed with poly(A)+ RNA from mixed blood stages (Fig. 1C). In good agreement with the previous expression profiling of *PfPlrx* [12], we detected the *PbPlrx* transcript in asexual blood stage parasites of the WT. As predicted, no *Plrx* transcripts were detected in the knockout parasite lines. Moreover, Western blot analysis of *Plrx(-)* blood stages with a *Pb*Plrx-specific anti-peptide antiserum confirmed complete absence of the protein in *Plrx(-)* parasites (Fig. 1D). Together, the successful generation of *Plrx*-deficient parasites demonstrates that this gene is not essential for proliferation of the asexual blood stages.

### *Plrx* is dispensable for *in vivo* and *in vitro* growth of asexual blood stages

**Figure 2:**
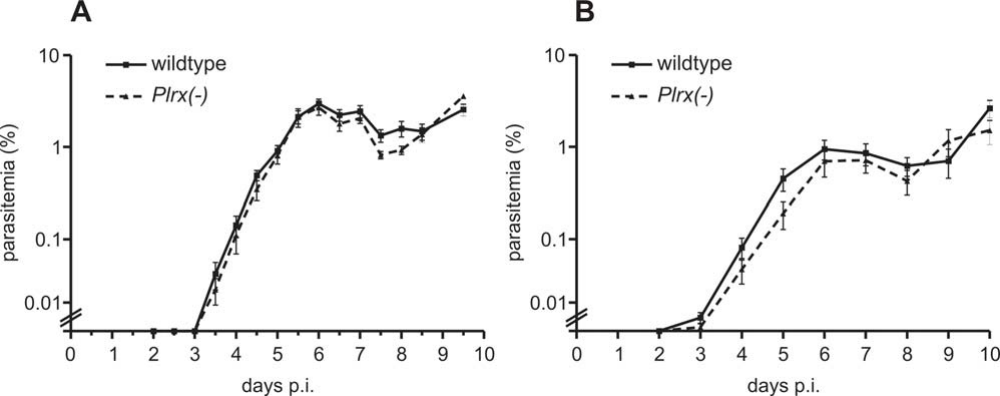
Replication of asexual blood stage parasites is unaffected in *Plrx(-)* mutant parasites. (A) *In vivo* growth curves of WT and *Plrx(-)* parasites. Five and six naïve animals were injected intravenously with 1,000 WT and *Plrx(-)* parasites, respectively. Parasitemia was determined every 12 hours after infection by microscopic examination of Giemsa-stained blood smears. (B) *In vivo* growth curves of WT and *Plrx(-)* parasites under constant exposure of methylene blue (50 mg/kg body weight). Treatment started immediately after infection with 1,000 WT and *Plrx(-)* parasites, respectively.

To test whether *Plrx* serves an auxiliary role during blood stage growth we first performed an *in vivo* growth assay by intravenous injection of 1,000 asexual parasites, followed by parasitemia counts every 12 hours post infection (Fig. 2A). Notably, proliferation of *Plrx(-)* parasites was indistinguishable from WT parasites indicating that *Plrx* is dispensable for the parasite under normal growth conditions. We next wanted to determine whether loss of *Plrx* function affects parasite growth under redox stress conditions. To this end, we performed an *in vitro* culture assay over 24 hours to determine the IC_50_ values in WT and *Plrx(-)* parasites upon exposure to antimalarial drugs, some of which act also as elicitors of redox stress [15]. The compounds tested included 4-aminoquinolines, i.e. chloroquine and amodiaquine, the first synthetic antimalarial agent methylene blue, mefloquine, and the central compound of artemisin-combination therapies (ACT) recommended as first-line antimalarial treatment, artemisinin. In order to minimize stage-specific effects we used non-synchronized asexual blood stages [16]. Notably, the IC_50_ values of the *Plrx(-)* parasite line did not differ significantly from WT parasites, excluding, at least *ex vivo*, a central function of *Plrx* in anti-redox stress defense (Table 1).

**Table 1:** *In vitro* characterization of *P. berghei* blood stages (IC_50_ data) [nM]

Drug	WT	*Plrx(-)*
Chloroquine	35.7 (±12.5)	33.4 (±6.3)
Methylene blue	50.6 (±9.6)	55 (±10.5)
Artemisinin	87.1 (±4.6)	79.2 (±23.5)
Mefloquine	50.1 (±21.1)	41.5 (±10.4)
Amodiaquine	20.5 (±10.4)	19.2 (±9.6)

To elucidate whether this dispensability can be observed *in vivo* we applied a growth assay as outlined above under enhanced oxidative stress conditions. We selected methylene blue (MB) as this antimalarial was shown to challenge the parasites intracellular reducing milieu through the generation of pro-oxidant H_2_O_2_ [17]. The WT and *Plrx(-)* infected mice were treated orally with 50 mg/kg body weight methylene blue as previous dose finding experiments revealed rapid elimination of WT parasites with 100 mg/kg body weight MB (data not shown). Even with enhanced oxidative stress *in vivo Plrx*-deficient parasites grow indistinguishable from WT parasites (Fig. 2B), excluding a central role of *P. berghei Plrx* in antioxidant defense.

### Depletion of *Plrx* induces only weak changes of transcript levels of selected redox proteins

**Figure 3:**
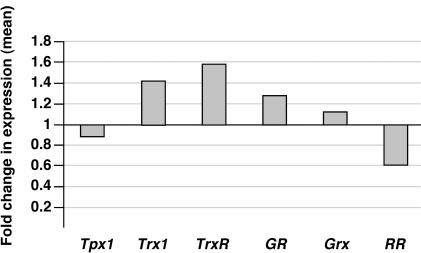
Depletion of *PbPlrx* reveals only weak alterations in gene expression of redox proteins. Gene transcript levels were measured by quantitative real-time RT-PCR. Cycle threshold and reaction efficiency of both the target gene and the reference gene (seryl-tRNA synthetase) were considered. The regulation of a target gene is reported as increase in comparison to the WT control population. Values represent mean values of three independent experiments. TPx-1: 2-Cys Peroxiredoxin, Trx1: Thioredoxin, TrxR: Thioredoxin Reductase, GR: Glutathione Reductase, Grx1: Glutaredoxin, RR: Ribonucleotide reductase.

Since our *in vitro* and *in vivo* data exclude an essential function of *Plrx* to maintain the parasite's redox equilibrium, we extended our analysis of the *Plrx*-deficient strain to expression profiling of selected redox proteins. This analysis was expected to further reveal the modulation of intracellular redox networks. We studied the effects of the *Plrx*-deletion on mRNA levels of genes related to cellular redox metabolism with a focus on the cytosolic components because *Plrx* is a cytosolic member of the antioxidant network [12]. Gene transcript levels were measured by quantitative real-time RT-PCR and the effect on a target gene is reported as differences in comparison to a WT control population (Fig. 3). Transcript levels of mRNA for the two major sustainers of redox homeostasis, thioredoxin reductase (*TrxR*) and glutathione reductase (*GR*) increased only slightly. *Plrx* was previously shown to directly interact with these two systems [12], thereby potentially acting as an additional antioxidant defence line of Plasmodium. The prediction was that deletion of *Plrx* may be accompanied by a compensatory upregulation of functional paralogues that balance the reducing capacity of *Plrx*. Such a function can most likely be fulfilled by thioredoxin (*Trx*) and/or glutathione (*GSH*). While this assumption is supported by the weak increase of *Trx* mRNA levels, *GSH* cannot be tested directly because it is only a tripeptide.

Another member of the thioredoxin superfamily, glutaredoxin (Grx) did not change significantly. Moreover, *Plrx*-deficient parasites show a slight decrease in mRNA levels of thioredoxin peroxidase 1 (*TPx1*), the major cytosolic peroxiredoxin of the parasite, and ribonucleotide reductase (*RiboR*). Collectively, these data show that depletion of *PbPlrx* caused only weak alterations in gene expression of selected members of the cytosolic redox network compared to wild type parasites.

### *Plrx* is not essential for completion of the *Plasmodium* life cycle

Since we could exclude a discernible function of *Plrx* in blood stage development we extended our phenotypic analysis to the entire *Plasmodium* life cycle (Table 2). *Plrx(-)* parasites did not differ from WT parasites in sexual development, which is a prerequisite for transmission to mosquitoes (data not shown). Dissection of infected mosquitoes showed similar numbers of oocysts, midgut-associated and salivary gland-associated sporozoites in WT and *Plrx* deficient parasites (Table 2). Therefore, Plrx is also dispensable for sporogony and sporozoite maturation. When mature salivary gland sporozoites were tested for infectivity to the mammalian host *in vivo* and *in vitro* again no phenotypic differences between the two parasite lines could be observed. Hepatocytes infected with *Plrx(-)* sporozoites were indistinguishable from WT infected cells and produced high numbers of mature liver stage parasites (Table 2). When tested *in vivo* by intravenous injection or natural mosquito bite the recipient animals became patent after similar prepatent periods compared to WT sporozoite inoculation (Table 2). Together these data exclude a vital role for *Plrx* in *Plasmodium* life cycle progression under standard conditions.

**Table 2:** Loss of *Plrx* function does not impair *Plasmodium* life cycle progression

		sporozoites			prepatency(days)^e^	
Parasite	Infectivity^a^	midgut^b^	salivary gland^c^	liver stages^d^	after i.v. injection	by bite
*Plrx(-)* f	74.1% (±9.7%)	17,100 (±27,200)	6,700 (±3,100)	344 (±1.4)	12/12 (d. 3.7)	10/10 (d. 5.7)
WT	88.8% (±1.8%)	13,900 (±10,300)	16,300 (±3,600)	N/D	8/8 (d. 3.8)	8/8 (d. 5.5)

a Percentage of mosquitoes that contain oocysts at day 11–13 post-feeding.

b Midgut-associated sporozoites per infected mosquito at day 12–14 post-feeding.

c Salivary gland-associated sporozoites per infected mosquito at day 17–19 post-feeding.

d Liver stages are mature exo-erythrocytic forms visualized 48 hours after incubation of 10,000 salivary gland sporozoites with subconfluent cultured hepatocytes.

e Prepatent period is the time until the first detection of an erythrocytic stage parasite in Giemsa-stained blood smears after intravenous injection of 10,000 salivary gland sporozoites (i.v.) or by bites of 5–10 infected mosquitoes.

f Four independent *Plrx(-)* clones (# 1,2,5, and 7) were fed. Shown are the average values from these phenotypically indistinguishable clones.

N/D, not done.

## Discussion

We initiated this study to test the potential of plasmoredoxin (Plrx), a *Plasmodium* specific member of the thioredoxin superfamily [12], as a novel antimalarial drug target. Using classical reverse genetics we could demonstrate that *Plrx* is dispensable for *Plasmodium* development inside its host cells. This finding rejects future drug discovery efforts that aim at specifically targeting Plrx, most likely even in combination with existing antimalarial drugs. Successful generation of *Plrx(-)* mutants permitted a detailed observation of the *in vivo* function of *Plrx* during life cycle progression of the malaria parasite. Again, no vital role at any stage of the parasite life cycle was revealed. Therefore, specific targeting of plasmoredoxin is not suitable either for transmission-blocking or causal-prophylactic malaria intervention strategies.

The redox-active proteins thioredoxin and glutaredoxin are founding members of the thioredoxin superfamily. Additional members include tryparedoxin of *Trypanosomes*, the protein disulfide isomerase and a few bacterial disulfide bondforming proteins. This group of proteins carries out oxidation and reduction reactions based on the chemistry of the catalytic cysteine residues. The unifying features of all family members are (i) the typical active site motif C-X-X-C and (ii) similarity in the overall tertiary protein structure, the so-called thioredoxin fold, despite low overall amino acid sequence similarity [18]. Intriguingly, homozygous deletions of the mouse cytoplasmic *Trx1* or the mitochondrial isoform *Trx2* resulted in early embryonic lethality indicating key roles of the thioredoxin system in the development of multicellular organisms [19], [20]. In analogy, the recent characterization of the *Plasmodium*-specific dithiol:disulfide oxidoreductase *Plrx* raised the attractive possibility that the protein performs an important function for the intracellular life style of the malaria parasite.

Thus far, five thioredoxin related proteins have been identified in *Plasmodium falciparum,* in addition to plasmoredoxin [21]. Cytosolic Trx1 is the major substrate of thioredoxin reductase. It reduces thioredoxin-dependent peroxidases and is also capable of reacting with peroxides, dehydroascorbate, lipoic acid, and lipoamide directly. Trx2 (and TPx2) were shown to be mitochondrial [22]. Trx2 also displays general disulfide reducing activity and serves as electron donor for thioredoxin peroxidises and GSSG. Trx3, which also carries a targeting sequence, has been shown to be redox active and reducible by *Pf*TrxR. In addition, two thioredoxin-like proteins, Tlp1, which might represent a small dynein subunit, and Tlp2 have been reported in *Plasmodium falciparum* and might exhibit partially overlapping functions with classical thioredoxins.

An explanation for the non-vital role of *Plrx* in malarial parasites might thus be redundancy in the function of the multiple members of the thioredoxin superfamily. There is precedence from other systems. For instance, the bacterial cytoplasm typically contains two thioredoxins, thioredoxin 1 (*trxA*) and thioredoxin 2 (*trxC*), as well as the glutaredoxin system comprising glutaredoxins 1 to 3 (*grxA-C*) [23], [24]. Out of these proteins three (*trxA*, *trxC*, and *grxA*) are able to reduce ribonucleotide reductase. Importantly, null mutants lacking any of these genes are viable [23], [25], whereas the corresponding *E. coli* triple knockout is not [26]. The combined vital role of these complementary bacterial redox proteins is best explained by their overlapping functions [27]. Therefore, in *E. coli* the presence of any one of these oxidoreductases is sufficient for survival [26]. Another example is found in *Saccharomyces cerevisiae* that encodes two cytoplasmic glutaredoxins (*Grx1/2*) [24], [28], [29] and two thioredoxins (*Trx1* and *Trx2*) [30]. In analogy to bacteria, only a quadruple yeast mutant is non-viable, and a single glutaredoxin or thioredoxin is able to restore viability [31]. However, strains deleted for glutaredoxins display altered sensitivity when exposed to various reactive oxygen species [28], [29]. In addition, the *Trx* double knockout displays a profound cell cycle defect, resulting in a prolonged S and a shortened G1 phase [30]. This effect again is presumably based on the inefficient ribonucleotide reduction, which results in insufficient supply of deoxyribonucleotides, a rate-limiting step in DNA synthesis. Due to advanced reverse genetic tools in yeast and *E. coli*, multiple knockouts of the GSH, glutaredoxin and thioredoxin pathways could be generated [26], [28]–[30] that established the overlapping nature of the two systems and provided crucial insight into the cellular responses to oxidative and reductive stress.

Currently, these genetic tools are not available for *P. berghei*. It will however be interesting to test in the future which redox-active proteins functionally overlap with *Plrx* in the malaria parasite. Plasmoredoxin may still fulfill specific and important functions in the cell in concert with other members of the thioredoxin superfamiliy that are not readily revealed under normal *in vivo* growth conditions. Under more pronounced stress conditions and in certain parasite stages these specific functions of plasmoredoxin might become evident. However, because of the non-vital role and the likelihood of major functional redundancy in *Plasmodium* redox defence and ribonucleotide reduction, *Plrx* can largely be excluded as a valid target for antimalarial intervention strategies. We propose that target validation by reverse genetics in the rodent malaria model parasites is an indispensable requirement for preclinical drug development.

## Materials and Methods

### Experimental animals

Sprague-Dawley rats, NMRI mice and C57bl/6 mice were obtained from Charles Rivers Laboratories. All animal experiments were conducted in accordance with European regulations and approved by the state authorities (Regierungspräsidium Karlsruhe).

### Drugs

The following drugs were used: chloroquine diphosphate and amodiaquine (Sigma-Aldrich, Steinheim, Germany), mefloquine-HCl (Roche, Mannheim, Germany), artemisinin (Aldrich Chemical Co., Milwaukee, Wis.) as well as methylene blue (Roth, Karlsruhe, Germany).

### Plasmoredoxin targeting vectors and *P. berghei* transfection

Two independent strategies, gene disruption and gene replacement, were used to disrupt the *P. berghei* plasmoredoxin gene using a standard *P. berghei* transfection vector, which contains the mutated *Toxoplasma gondii dhfr/ts* gene as a marker for positive selection with the antifolate pyrimethamine [32]. The *Plrx* integration vector was generated by combining two PCR fragments that were amplified using the following primer pairs and *P. berghei* genomic DNA as template: *Pb*PlrxInt1 for (5′-CGGGATCCCATTCTACCCAAAATGAAGCACCC-3′; *Bam*HI site is underlined and *Pb*PlrxInt1 rev (5′- GGACTAGTATATATATTATTTCAAAAAAGGG-3′; *Spe*I site is underlined); *Pb*PlrxInt2 for (5′-GGACTAGTTGATCAAACATATACAGATTATATCAATT-3′; *Spe*I site is underlined) and *Pb*PlrxInt2 rev (5′- TCCCCGCGGCGATTATTTGATTTGAATGTTTTGGGG-3′; *Sac*II site is underlined). For integration of the targeting vector via single cross-over the introduced cleavage site (*Spe*I) was used. The *Plrx* replacement vector was generated using the primers *Pb*Rep1 for (5′- GGGGTACCCAGCATTGATAACAGTATGAACAACAGC-3′; *Kpn*I site is underlined) and *Pb*Rep1 rev (5′-CCCAAGCTTTACGAAGAACTTAACAAAGCTCATCG-3′; *Hin*dIII site is underlined); *Pb*Rep2 for (5′- GGACTAGTGTTATGTTGTTGCATCCTAAACCTCAAAC-3′; *Spe*I site is underlined) and *Pb*Rep2 rev (5′-TCCCCGCGGCTCGTTGGATGATTTTGAAATTGCC-3′; *Sac*II site is underlined). Sequence analyses confirmed the correct sequence of the different plasmids. *P. berghei* transfection and positive selection was done by the Nucleofector technology [33]. Clonal parasites were obtained by limited dilution of single parasites into recipient NMRI mice. To test for proper replacement of the *Plrx* gene, integration-specific primer combinations, Tgfor (5′-CCCGCACGGACGAATCCAGATGG-3′) and Plrxtestrev (5′- GGAACGTTTGCTACTCC-3′), as well as 5′testfor (5′- GCCAATGTACCCATGTACACAGC-3′) and Tgrev (5′- CCAACTCAATTTAATAGATGTGTTATGTG-3′) were used. The resulting PCR products were sequenced to verify the correct gene replacement and the accurate generation of the recombinant *Plrx(-)* locus. To test for the presence of residual WT parasites a *Plrx*-specific primer pair, Plrxstart (5′- ATGGCATGTAAAGTTGATAAAGC-3′) and Plrxend (5′- CTAATTGTAGAATAAATCGAAAAATCG-3′), was used. For the control amplification a glutathione reductase-specific primer pair, *Pb*GRfor (5′- TTGCGTAAATGTAGGTTGTGTACC-3′) and *Pb*GRrev (5′- AGTCAAGATGCTCAAACTTCCG -3′), was tested. We obtained four independent *Plrx(-)REP* clonal parasite populations that were phenotypically identical. Detailed analysis was performed with one representative clone.

### Immunoblotting

Three rats were infected with WT and *Plrx(-)* blood stage parasites, respectively, and cytosolic parasite proteins were extracted using 2M Urea buffer. For immunoblot analysis, proteins were separated on 15% polyacrylamide gels and transferred to nitrocellulose membranes (Bio-Rad) by electroblotting. Plrx (21.5 kDA) was detected by incubation of membranes with a polyclonal anti-*P. berghei* Plrx antiserum (dilution 1:1,500). This antiserum was obtained by immunization of rabbits with synthetic peptides of the N-terminal region of Plrx (Plrx-1: CYYKNNELKKIDSSYFQDKY and Plrx-2: CKVDKALEHSTQNEAPSK) (Eurogentec, Seraing, Belgium). Bound antibodies were detected using peroxidase coupled anti–rabbit and anti–mouse antibodies, to detect *Pb*Plrx and actin, respectively. Immunostained proteins were visualized with enhanced chemoluminescence detection (Pierce). Recombinant *P. berghei* Plrx was used as a positive control (obtained according to [12]). The anti-*Dictyostelium discoideum* actin antiserum, which cross-reacts with apicomplexan actins, was kindly provided by Dr. Markus Meissner (Heidelberg).

### Determination of IC_50_ values

NMRI mice were infected with *P. berghei* WT and *Plrx(-)* parasites. Collection of infected erythrocytes was done by cardiac puncture. Infected erythrocytes were cultured in an isotopic drug sensitivity assay based on incorporation of radioactive [3H] hypoxanthine (protocol kindly provided by Sergio Wittlin, Basel, Switzerland). Briefly, serial dilutions of various drugs were prepared in 96-well microtiter plates (Nunc). 100 µl infected red blood cells, resulting in 5% hematocrit and 1-5% parasitemia, were added per well and incubated in hypoxanthine-free medium at 37°C in a gas mixture consisting of 94% N_2_, 3% O_2_ and 3% CO_2_. After 16 h incubation, 0.5 µCi of [3H] hypoxanthine (Amersham Pharmacia) in 50 µl medium was added and plates were incubated for an additional 8 h. Parasites were harvested onto glass fiber filters (Perkin-Elmer, Rodgau-Jügesheim, Germany), washed and dried. Radioactivity was counted using a β-counter (Matrix 9600; Packard). The results were recorded as counts per minute (cpm) per well at each drug concentration and growth inhibition was expressed as percent 3H incorporation compared with untreated controls. Fifty percent inhibitory concentrations (IC_50_) were calculated from plotted data. All experiments were done in duplicate from at least three independent mouse infections each.

### Expression profiles of *Plasmodium* WT and *Plrx(-)* parasites using quantitative real-time RT-PCR

For qRT-PCR analyses poly(A)^+^ RNA was isolated using oligo(dT) columns (Macherey-Nagel, Düren, Germany) from mixed blood stage of *Plasmodium berghei* WT and *Plrx(-)* lines. After a DNase treatment, aliquots of 800 ng of each sample were reversely transcribed to cDNA using an Abgene cDNA synthesis kit and oligo(dT) primers (Abgene, Hamburg, Germany). The SYBR Green Jumpstart Taq Ready Mix (Sigma, Steinheim, Germany) was utilized for quantitative real-time PCR on Rotor-Gene 3000 Real-Time PCR system (Corbett Research, Sydney, Australia) using the primers listed in Table 3. Specificity of the amplification products was confirmed by melting curve analysis; a no-template control was added in every run. The Rotor-gene 6.0 software was used to retrieve the real time PCR results, to analyse the data and to determine cycle threshold values. Relative expression of target genes were calculated based on efficiency (determined with dilution series of each gene and the Rotor-Gene software) and Ct-values for genes of Plrx-deficient parasites versus control WT-parasites, and expressed in comparison to a reference gene. The relative amount of mRNA was determined according to the Pfaffl equation [34], using *P. berghei* seryl-tRNA synthetase as the reference gene. In our experiments, each RT-PCR run was carried out in triplets of one mRNA pool or in doublets of two different mRNA pools and the whole series was reproduced in an independent experiment.

**Table 3:** Oligonucleotide primers used for the quantitative real time PCR.

Primer name	Sequence	Product Size (bp)
Pb stRNA^a^ s	TAGTGGCTGGACATAGAGGTT	158 bp
Pb stRNA^a^ as	CTCTGCACATTCTTCCATTA	
Pb GR^b^ s	CTTGCGTAAATGTAGGATGTG	138 bp
Pb GR^b^ as	CTCTCCTTTCTACTAATTGTG	
Pb TrxR^c^ s	ATGGATTAGCAAAATTAAAAAAT	125 bp
Pb TrxR^c^ as	CTGGTATATTTGGTCGACATCC	
Pb Grx1^d^ s	GAAAATAAAATTGCTGTATTCTC	149 bp
Pb Grx1^d^ as	TTGAAATATGATTGGATATCTGCC	
Pb Trx1^e^ s	TGGACCTTGCAAAAGAATAGCTCC	197 bp
Pb Trx1^e^ as	TCAAAGCTCCCTCATTGGCTCC	
Pb TPx-1^f^ s	TAGATAAACAAGGAGTTGTTC	160 bp
Pb TPx-1^f^ as	TCCGATGGTTTCATGGATTCC	
Pb RiboR^g^ s	GCTGATAGATTACTAGAATGTTTAGGG	165 bp
Pb RiboR^g^ as	GCCATAACACCTGACTTTTGATAATCTGC	

a stRNA: seryl-tRNA synthetase,

b GR: Glutathione Reductase,

c TrxR: Thioredoxin Reductase,

d Grx1: Glutaredoxin,

e Trx1: Thioredoxin,

f TPx-1: 2-Cys Peroxiredoxin,

g RiboR: Ribonucleotide reductase; s : sense primer, as : antisense primer

### *Plasmodium* life cycle and phenotypic analysis of *Plrx(-)* parasites

*Anopheles stephensi* mosquitoes were kept at 21°C, 80% humidity and daily feeding on 10% sucrose. Asynchronous blood-stages of *P. berghei* NK65 (WT) and *Plrx(-)* were maintained in NMRI mice and checked for gametocyte formation and exflagellation of microgametes prior to mosquito feeding. For mosquito infection, *A. stephensi* mosquitoes were allowed to bloodfeed on anesthetized mice for 15 minutes. Dissection of mosquitoes was conducted at days 10, 14 and 17 in order to determine infectivity and sporozoite numbers in midguts and salivary glands, respectively. Gliding motility was assessed by deposition of sporozoites onto precoated glass coverslips and visualisation by indirect immunofluorescence using a primary antibody against the *P. berghei* circumsporozoite protein (CSP) [35] followed by detection with an Alexa Fluor 488-conjugated anti-mouse antibody. To analyse liver stage development sporozoites were deposited onto a semi-confluent monolayer of hepatoma cells (HuH7) and incubated for 2 h, followed by washing and incubation in cell culture medium. Liver stages were detected after 48 h with a primary antibody directed against the *P. berghei* heat shock protein 70 (HSP70) [36], followed by an Alexa Fluor 488-conjugated anti-mouse antibody. To analyse sporozoite infectivity *in vivo*, Sprague-Dawley rats were infected intravenously with 10,000 WT or *Plrx(-)* sporozoites, respectively. Parasitemia was followed by daily examination of Giemsa-stained blood smears. The occurrence of a single parasite marked the first day of patency.
